# Structurally optimized honeycomb scaffolds with outstanding ability for vertical bone augmentation

**DOI:** 10.1016/j.jare.2021.12.010

**Published:** 2022-01-05

**Authors:** Koichiro Hayashi, Masaya Shimabukuro, Ryo Kishida, Akira Tsuchiya, Kunio Ishikawa

**Affiliations:** Department of Biomaterials, Faculty of Dental Science, Kyushu University, 3-1-1 Maidashi, Higashi-ku, Fukuoka 812-8582, Japan

**Keywords:** Honeycomb, Scaffold, Dental implants, Apatite, Vertical bone augmentation

## Abstract

**Introduction:**

Cases of intractable dental implant require vertical bone augmentation; however, the predicted bone height and volume are difficult to obtain. In vertical bone augmentation, the contact surface between the scaffold and the bone is limited to the bottom face of the scaffold. Furthermore, the strength decrease caused by scaffold resorption leads to collapse of the augmented site, leading to a decrease in the bone volume and height.

**Objectives:**

To promote bone ingrowth, we fabricated carbonate apatite (i.e., bone mineral) honeycomb (HC) scaffolds with uniaxial channels vertically penetrating the scaffold. Furthermore, we controlled the scaffold resorption rate, eventually the endurability for compression, and the bone height and volume by controlling the strut thickness.

**Methods:**

The channel aperture was controlled to be 230–260 μm to promote bone ingrowth. Furthermore, the strut thicknesses of the HC scaffolds were adjusted to 100, 200, and 300 μm to control the scaffold resorption; these scaffolds were designated as HC100, HC200, and HC300, respectively.

**Results:**

At 4 weeks post-implantation on rabbit calvarium, all scaffolds had already vertically augmented new bone close to the top surface of the scaffold. In the following 8 weeks, the height and amount of new bone in all scaffolds increased. Notably, HC300 was resorbed synchronously with new bone formation, allowing it to endure the compression from the fasciae for 12 weeks post-implantation. Furthermore, HC300 formed larger-diameter blood vessels than those of HC100 and HC200.

**Conclusion:**

The HC scaffolds surpassed the various combined scaffolds and growth factors or stem cells in the ability for vertical bone augmentation. Thus, the HC structure is inherently suitable for vertical bone augmentation. Notably, the HC scaffolds with 300-μm-thick struts enhanced both new bone formation and angiogenesis. This study revealed a structurally suitable design for achieving an outstanding outcome in vertical bone augmentation.

## Highlights

• We fabricated carbonate apatite honeycomb scaffolds with vertical uniaxial channels.
• At 4 weeks post implantation, all honeycomb scaffolds augmented new bone.
• The augmentation happened close to the top surface of the scaffold.
• In the following 8 weeks, the height and amount of new bone increased.
• HC300 resisted compression from fasciae at 12-weeks post implantation.
• Honeycomb structure is inherently suitable for vertical bone augmentation.

## Graphical abstract

**Figure:**
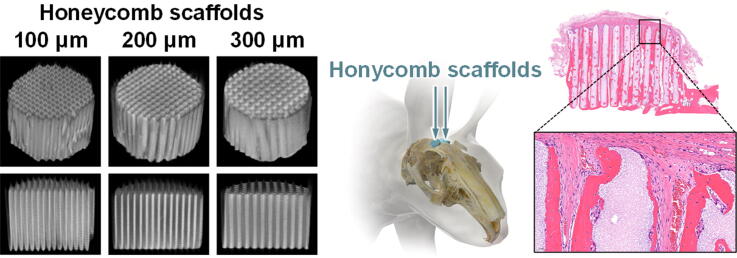

## Introduction

Alveolar ridge deficiencies prevent the long-term success of dental implants [1], [2], [3], [4], [5], [6]. Therefore, bone augmentation is imperative when alveolar bone undergoes advanced resorption [1], [2], [3], [4], [5], [6]. Among various bone defects, vertical bone defects are the most difficult to augment [7], [8]. Autogenous bone graft is still the gold standard for vertical bone augmentation as it exhibits osteoconductive and osteoinductive properties [3]. However, the harvestable quantity of autogenous bone is limited and invasive surgery is ineluctable [9], [10], [11], [12], [13]. Furthermore, autologous bone is commonly associated with rapid resorption owing to mechanical pressure from soft tissues in the recipient sites [11], [14]. Some reports indicate that approximately 40% of the graft resorbs during the early phase [15], [16]. The rapid resorption of autogenous bone often leads to the collapse of the augmented site, thereby leading to a decrease in the bone volume and height way below expectation, eventually resulting in failure of the subsequent implant placement [11], [14], [15], [16].

Guided bone regeneration using barrier membranes is also a common technique for vertical bone augmentation [17], [18], [19]. The barrier membranes function by creating space above the bony defect and preventing the invasion of faster-migrating connective tissues and epithelial cells into the space, allowing the ingrowth of bone into the space [17], [18], [19]. However, the barrier membranes also hinder angiogenesis from the subperiosteum [20]. Consequently, osteogenesis and angiogenesis rely solely on the basal bone outside any bony envelope [20]. Thus, to ensure the success of vertical bone augmentation, the development of scaffolds that facilitate the ingrowth of bone and blood vessels from the basal bone is deemed effective for maintaining the height and volume of newly formed bone. Although various calcium phosphate scaffolds have been developed, they are not efficient in bearing the compression from soft tissues, resulting in deformation or collapse [21], [22], [23]. This is likely caused by a decrease in the compressive strength of the scaffold, which is associated with scaffold resorption. An effective strategy to prevent it is to induce the formation of new bone simultaneously or immediately after scaffold resorption and compensate the decreased compressive strength with the new bone. In order to achieve this, it is necessary to improve the scaffold ability to vertically augment the new bone. To improve such ability, scaffolds have been combined with growth factors [24], bone morphogenetic protein (BMP) [25], [26], [27], and progenitor and/or stem cells [28], [29], [30], [31], [32]. However, these methods have various limitations, such as the formation of severe cyst-like tissues and swelling, excess bone resorption by osteoclasts, elaborate procedures, high cost, and/or invasive manipulation that requires surgeries at least twice [33], [34], [35]. Therefore, ideally, the synchronization of scaffold resorption and new bone formation should be achieved by merely controlling the scaffold structure, without use of growth factors, BMP, or cells; in this way, the scaffold would be replaced by new bone while bearing compression from the fasciae.

Honeycomb (HC) scaffolds have the potential of achieving the abovementioned requirements owing to the following reasons: (1) their resorption rate and mechanical strength can be controlled by adjusting strut thickness [36]; (2) their uniaxial channels facilitate the ingrowth of bone and blood vessels (channels with 230–300 μm opening size are more effective than smaller and larger channels) [36], [37], [38]; (3) micropores (<2 μm) within the struts of HC scaffolds promote bone formation [39], [40], [41]; and (4) predictably sized micropores can be created within the struts by constructing the struts with spherical components based on sphere packing theory: packing 5 μm spheres creates micropores <1 μm, resulting in the enhancement of osteogenesis and angiogenesis [37], [38], [41]. Notably, HC scaffolds consisting of carbonate apatite, which is analogous to a bone mineral, show a higher osteoconductivity than those consisting of β-tricalcium phosphate and hydroxyapatite [42], [43]. Furthermore, carbonate apatite HC scaffolds exhibit osteoinductivity as well as osteoconductivity [44].

Considering these characteristics of carbonate apatite HC scaffolds, we may achieve vertical bone augmentation as predicted by strut thickness control that manages the resorption rate and mechanical strength of HC scaffolds. Herein, we demonstrate the correlation of the strut thickness of HC scaffolds with their resorption rate, endurability for compression from soft tissues, and the height and volume of the newly augmented bone.

## Material and methods

### Ethics statement

All experiments involving animals were conducted according to the ethical policies and procedures approved by the Animal Care and Use Committee of Kyushu University, Japan (Approval no. A21-307-0; issued May 17, 2021).

### Materials

CaCO_3_ powder was purchased from Sakai Chemical Industry (Osaka, Japan), methylcellulose-based binder from Matsumoto Yushi-Seiyaku (Osaka, Japan), and Na_2_HPO_4_ from FUJIFILM Wako Pure Chemical Corporation (Osaka, Japan).

### Fabrication of carbonate apatite HC scaffolds

HC scaffolds were fabricated based on extrusion molding and the spherical packing theory [37]. In short, a mixture of CaCO_3_ powder and a methylcellulose-based binder was extruded through dies with slit thicknesses of 100, 200, and 300 μm and pitch of 400, 500, and 600 μm using a vacuum extruder (V-30-II, Universe C., Saga, Japan). HC green bodies with struts of approximately 100, 200, and 300 μm in thickness and channels of approximately 300 μm in opening size were obtained. These HC green bodies were heated at 600 °C for 24 h in an electric furnace (DMT-01, Nidec-Shimpo, Kyoto, Japan) for the removal of organic binder, resulting in the formation of CaCO_3_ HC blocks. The CaCO_3_ HC blocks were immersed in 1 mol/L Na_2_HPO_4_ solution at 80 °C for 7 days. Consequently, carbonate apatite HC scaffolds were formed through dissolution–precipitation reactions. The carbonate apatite HC scaffolds using dies with slit thicknesses of 100, 200, and 300 μm were designated as HC100, HC200, and HC300, respectively. Finally, these HC scaffolds were precisely shaped into cylinders of 6-mm diameter and 4-mm height by computer-controlled machining using a 3D milling machine (monoFab SPM-20, Roland DG, Shizuoka, Japan).

### Physicochemical and mechanical characteristics of HC scaffolds

The X-ray diffraction patterns of the HC scaffolds were measured to determine the crystal phases using a X-ray diffractometer (XRD; D8 Advance, Bruker AXS GmbH, Karlsruhe, Germany). Hydroxyapatite (Taihei Chemical Industrial, Osaka, Japan) was used as the reference. The functional groups in the HC scaffolds were determined using a Fourier transform infrared (FT-IR) spectrophotometer (FT/IR-6200, JASCO, Tokyo, Japan). The percentages of carbonate contained in the HC scaffolds were measured using an elemental CHN analyzer (MT-6, Yanako Analytical Instruments, Kyoto, Japan). The average percentages of carbonate contained in the HC scaffolds were calculated from the values of eight samples per group.

The appearances and cross-sectional structures of the HC scaffolds were visualized by μ-CT imaging (SkyScan, Bruker Corporation, Billerica, MA, USA). The microstructures of the HC scaffolds were observed using a scanning electron microscope (SEM; S3400N, Hitachi High-Technologies, Tokyo, Japan).

The porosities of the HC scaffolds were calculated from the bulk density and the theoretical density of hydroxyapatite (3.16 g/cm^3^), as the theoretical density of carbonate apatite is unknown. The average porosities of the HC scaffolds were calculated from the results of eight samples per group.

The mechanical strengths of the HC scaffolds were measured by uniaxial compression test using a universal testing machine (Autograph AGS-J, Shimadzu Corporation, Kyoto, Japan). The average compressive strengths of the HC scaffolds were calculated from the results of eight samples per group.

The degradation of HC300 was estimated by evaluating its dissolution behavior in physiological saline (pH = 7.3) and a weak acid solution (pH = 5.5) corresponding to the condition during osteoclastic resorption. These evaluations were conducted in accordance with a method defined in Japanese Industrial Standards T0330-3; Bioceramics -Part 3: Testing method of measuring dissolution rate of calcium phosphate ceramics. In brief, HC300 was immersed in 50 mmol L^−1^ physiological saline (pH = 7.3) or 80 mmol L^−1^ acetic acid sodium acetate buffer solution (pH 5.5) at 25 ± 3 °C. The supernatant was collected and diluted (hundredfold). The calcium ion concentration in the diluted supernatant was measured using inductively coupled plasma optical emission spectrometry (Optima 7300 DV; PerkinElmer, MA). The content of calcium ions in HC300 was measured by completely dissolving HC300 in nitric acid solution and measuring calcium ion concentration in the solution using the same method (n = 5).

### In vitro alkaline phosphatase (ALP) activity assay

MC3T3-E1 cells (2 × 10^4^ cells) were seeded on HC300 (n = 5) in 24-well plates and cultured in alpha-minimal essential medium containing 10% fetal bovine serum, streptomycin (100 μm mL^−1^; FUJIFILM Wako Pure Chemical Corporation), penicillin (100 U mL^−1^; FUJIFILM Wako Pure Chemical Corporation), and amphotericin B (0.25 μg mL^−1^; FUJIFILM Wako Pure Chemical Corporation). The culture medium was changed every 3 days. At days 7 and 14 of culture, the cells were washed thrice with phosphate-buffered saline (PBS; FUJIFILM Wako Pure Chemical Corporation) and they were lysed with cell lysis buffer. Then, ALP activity was assessed using an ALP assay kit (FUJIFILM Wako Pure Chemical Corporation) according to the manufacturer’s instructions. Absorbance at 405 nm was measured using a microplate reader (Multiskan FC; Thermo Fisher Scientific, MA, USA).

### In vitro Alizarin red staining

At day 21 of cell culture, HC300 was collected from the medium and washed with PBS five times. Calcified depositions formed on HC300 were stained with Alizarin Red S (FUJIFILM Wako Pure Chemical Corporation). Pure HC300 before cell seeding was stained with Alizarin Red S as a control.

### Animals

Japanese white rabbits (18 weeks of age, 3.0–3.5 kg of body weight) were purchased from Japan SLC (Shizuoka, Japan) for in vivo evaluations. These rabbits were single-housed in cages and maintained on a standard diet with an adequate amount of water in the Center of Biomedical Research, Research Center for Human Disease Modeling, Graduate School of Medical Sciences, Kyushu University. In total, 18 rabbits were used (n = 6 per group).

### Surgical procedure

In brief, the rabbits were subjected to an intraperitoneal injection of anesthesia composed of combined xylazine (5.0 mg/kg) and ketamine (30 mg/kg). The head fur of the rabbit was shaved and the skin was disinfected with 10% w/v povidone–iodine (Meiji Seika Pharma, Tokyo, Japan). The calvarium was exposed by making an incision in the head skin (approximately 2 cm in length) using a scalpel. The periosteum was separated from the bone using a raspatorium. Two HC scaffolds in each animal were implanted randomly on the calvarium. The top surfaces of the HC scaffolds were covered with fasciae, and the fasciae were sutured. Finally, the incised skin was sutured and the surgical site was disinfected with 10% w/v povidone–iodine.

### Radiographic analyses

At 4 and 12 weeks after the implantation of the HC scaffolds, the rabbit calvarium (n = 6 per group) was collected and fixed with formalin for evaluation of the resorption rate of the HC scaffolds and their bone formation abilities by radiographic and histological analyses. Volumetric analyses of all specimens were carried out using images obtained by μ-CT scanning (SkyScan, Bruker Corporation). The volume percentages of newly formed bone within the HC scaffold and the remaining scaffold were measured using a CT Analyzer software.

### Histological analyses

After μ-CT scanning of the specimens, these were decalcified, embedded in paraffin, and cut to obtain serial longitudinal sections of the HC scaffold. The sections were treated with hematoxylin and eosin (HE), Masson’s trichrome (MT), and CD31 antibody staining. Regions stained blue by MT corresponded to collagen fibers in the bone and vascular endothelium. Vascular endothelial cells were detected by CD31 antibody staining.

For CD31 antibody staining, deparaffinized sections were subjected to antigen retrieval using 20 μg/mL proteinase K solution. The sections were washed with tris-buffered saline (TBS), blocked with skim milk, and then treated with the monoclonal mouse anti-human CD31 antibody (DAKO M0823), which reacts with the rabbit antigen. Then, the sections were washed with TBS and incubated with horseradish peroxidase-conjugated secondary antibodies using diaminobenzidine as a chromogen.

The histological images of the HE-, MT-, and CD31-stained tissue sections were obtained using a biological fluorescence microscope (BZ-X, Keyence, Osaka, Japan). The height of newly formed bone and the area percentages of the remaining scaffolds and newly formed bone in each scaffold channel and the entire scaffold were estimated by analyzing HE-stained sections with BZ-X digital analysis software (Keyence). The diameters of blood vessels formed in the scaffold were estimated using CD31-stained sections with the BZ-X digital analysis software.

### Statistical analysis

All data are presented as mean ± standard deviation. The *p*-values < 0.05 were considered statistically significant. Comparisons between groups were performed using the Tukey–Kramer test.

## Results

### Scaffold characteristics

**Fig. 1:**
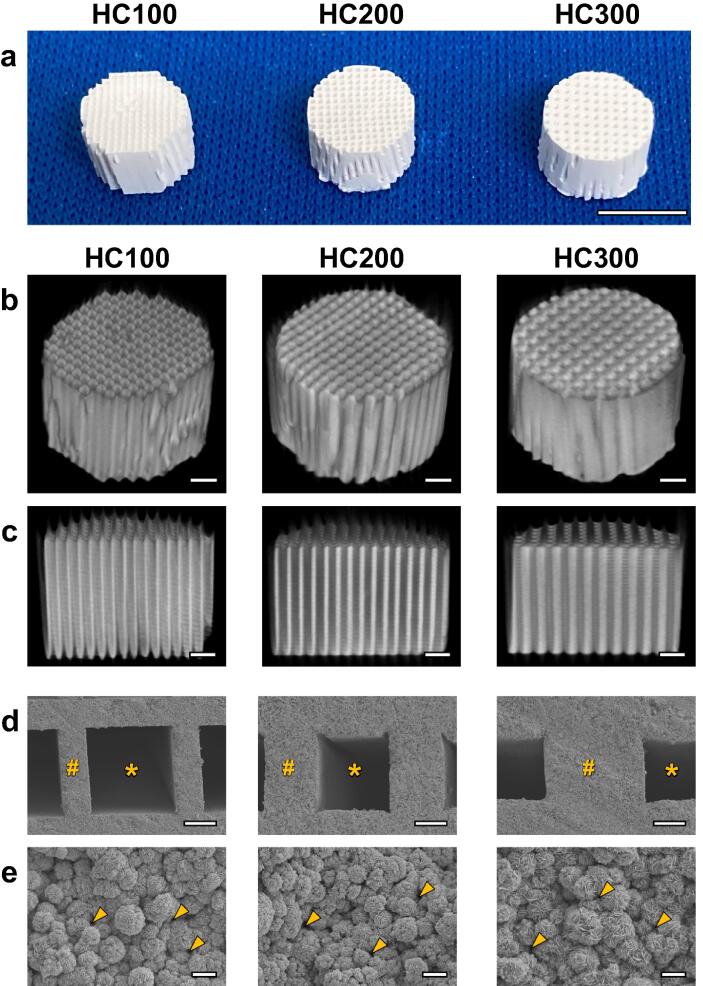
Photographs (a), μ-CT images (b and c), and scanning electron micrographs (d and e) of HC100, HC200, and HC300. The panel c displays longitudinal sections of the panel b. The panel e is the magnified view of the struts in panel d. “*,” “#”, and arrow heads indicate channel, strut, and micropores, respectively. Scale bars: 5 mm in panel a, 1 mm in panels b and c, 100 μm in panel d, and 5 μm in panel e.

HC100, HC200, and HC300 had an HC structure in which the struts and channels were orderly arranged (Fig. 1a and 1b). The uniaxial channels with an opening size of 230–260 μm vertically penetrated these HC scaffolds (Fig. 1c). The strut thickness in HC100, HC200, and HC300 was 99 ± 1, 190 ± 4, and 306 ± 3 μm, respectively (Fig. 1d). Thus, the strut thickness was approximately equal to the slit size of each die, indicating that the HC scaffold structure was controllable by the die design. The struts were constructed by interconnecting microspheres and micropores <1 μm (Fig. 1e).

The XRD patterns confirmed that HC100, HC200, and HC300 were composed of apatite crystals (Fig. S1a). The FT-IR spectra showed absorption bands attributed to phosphate at 1158–954 and 611–539 cm^−1^ for these HC scaffolds and hydroxyapatite, respectively (Fig. S1b) [45], [46]. Although the absorption band corresponding to hydroxyl was present at 631 cm^−1^ in the spectrum of hydroxyapatite, it was absent in the spectra of the HC scaffolds [45], [46]. Doublet bands attributed to carbonate appeared at 1477 and 1414 cm^−1^ in the spectra of the HC scaffolds, whereas these were not detected in the spectrum of hydroxyapatite [45], [46]. The disappearance of the hydroxyl band and appearance of the doublet carbonate bands in the spectra of the HC scaffolds corroborated that carbonate replaced both hydroxyl and phosphate in the apatite crystal [45], [46]. Thus, all these HC scaffolds were composed of AB-type carbonate apatite [46]. Furthermore, the CHN elemental analysis confirmed that the carbonate contents in these HC scaffolds were 8.5–10.5%, which is equal to that in human bone [47].

**Fig. 2:**
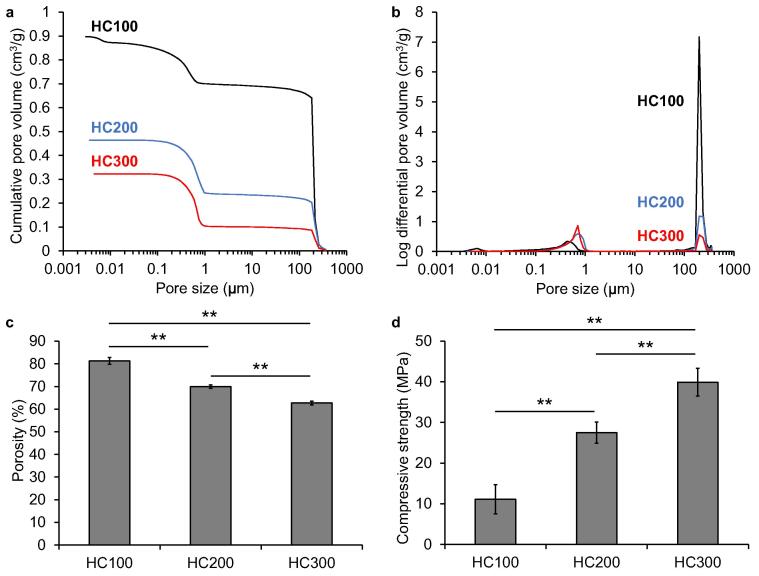
Results of mercury intrusion porosimetry. Pore size distribution and cumulative pore volume (a) and (b) pore diameter of HC100, HC200, and HC300. Porosities (c) and compressive strengths (d) of HC100, HC200, and HC300; ^**^p < 0.01.

**Fig. 3:**
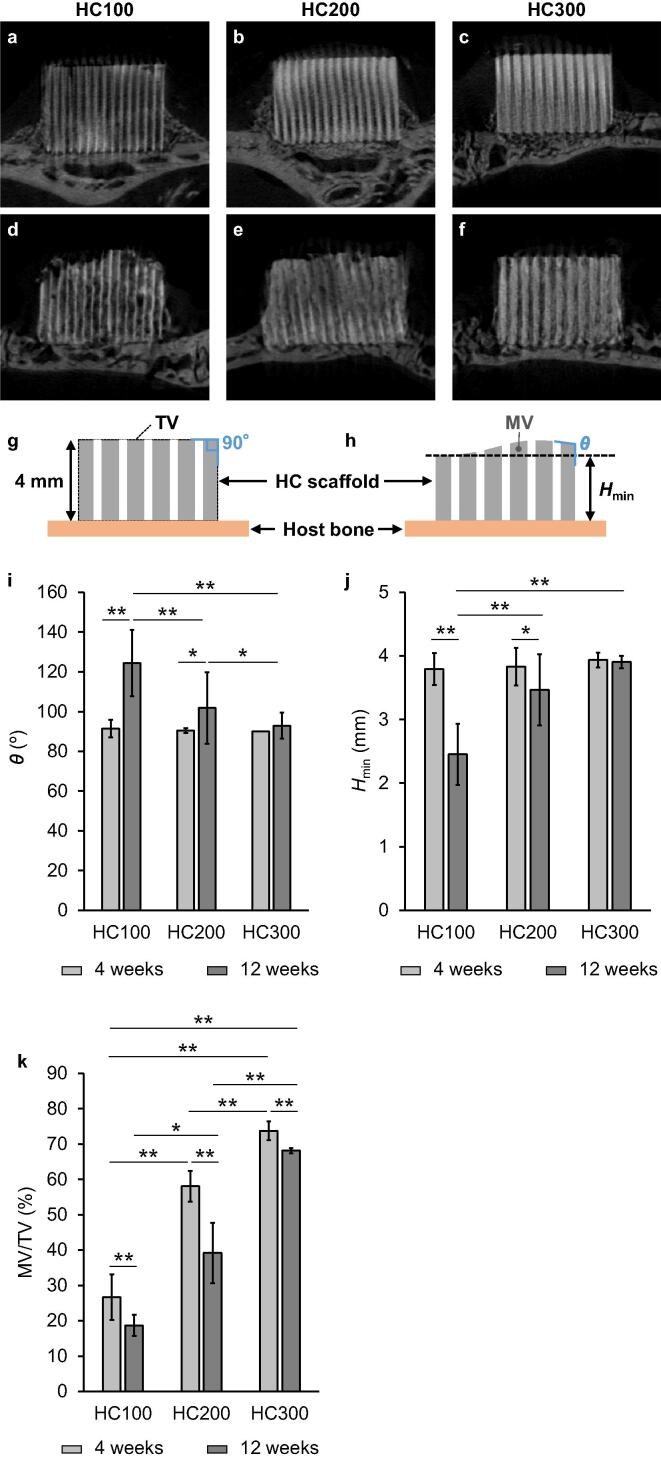
μ-CT images of HC100 (a), HC200 (b), and HC300 (c) at 4 weeks post-implantation; HC100 (d), HC200 (e), and HC300 (f) at 12 weeks post-implantation. Illustration of the honeycomb (HC) scaffolds before (g) and after (h) implantation; *θ* and *H*_min_ indicate the angle of side edges of the top surface and the minimum height of HC scaffold after implantation, respectively. TV and MV indicate the total volume of the HC scaffold (i.e., the sum of channel volume and strut volume) before implantation and the material volume (i.e., the volume of remaining struts) after implantation, respectively. The *θ* (i), *H*_min_ (j), and MV/TV (k) at 4 weeks and 12 weeks PI. *p < 0.05 and ^**^p < 0.01.

The results of mercury intrusion porosimetry in HC100, HC200, and HC300 showed bimodal peaks at approximately 200 μm and 0.1–1 μm (Fig. 2a), which corresponded to the opening size of the channels and the micropore size within the struts (Fig. 1), respectively. The volumes of channels in HC100, HC200, and HC300 were 0.70, 0.24, and 0.10 cm^3^/g, respectively (Fig. 3b), which coincided with an increase in the number of channels in the HC scaffold. The volumes of micropores in HC100, HC200, and HC300 were 0.20, 0.22, and 0.22 cm^3^/g, respectively (Fig. 2b), indicating that the HC scaffolds contained almost equal volumes of micropores in the struts. The porosities of HC100, HC200, and HC300 were 81.3 ± 1.1%, 65.9 ± 1.5%, and 62.7 ± 0.6%, respectively (Fig. 2c). The compressive strengths of HC100, HC200, and HC300 were 11.1 ± 3.3, 27.5 ± 3.6, and 39.9 ± 5.0 MPa, respectively (Fig. 2d). The porosity and compressive strength significantly decreased and increased with increasing strut thickness, respectively (*p* < 0.01).

**Fig. 4:**
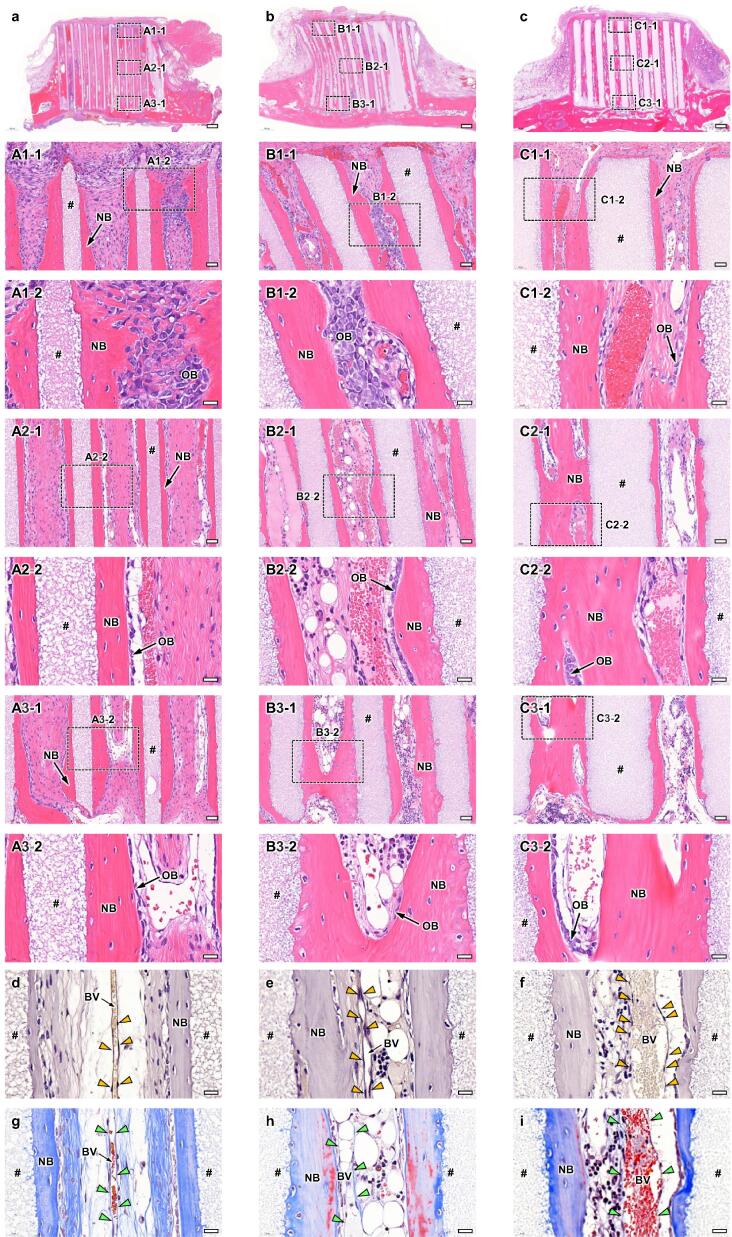
HE-stained sections at 4 weeks PI of HC100 (a), HC200 (b), and HC300 (c). Overviews (a-c); scale bars, 500 μm. Magnified images (A1-1-C1-1, A2-1-C2-1, and A3-1-C3-1); images of the regions A1-1-C1-1, A2-1-C2-1, and A3-1-C3-1 shown in images a-c; scale bars, 50 μm. Higher magnified images (A1-2-C1-2, A2-2-C2-2, and A3-2-C3-2); images of the regions A1-2-C1-2, A2-2-C2-2, and A3-2-C3-2 shown in images A1-1-C1-1, A2-1-C2-1, and A3-1-C3-1; scale bars, 20 μm. “#,” “NB,” and “OB” indicate strut, new bone, and osteoblast, respectively. CD31-stained sections of HC100 (d), HC200 (e), and HC300 (f); scale bars: 20 μm. Yellow arrowheads indicate vascular endothelial cells. “BV” indicates blood vessel. MT-stained sections of HC100 (g), HC200 (h), and HC300 (i); scale bars: 20 μm. Green arrowheads indicate vascular endothelium.

The above findings demonstrated that the characteristics of the channels and micropores were almost equal among HC100, HC200, and HC300, and only the strut thickness was different among these HC scaffolds. Furthermore, these characteristics of the channels and micropores are identical to those optimized in our previous studies [36–41]. Thus, the use of these HC scaffolds was found to be appropriate for evaluating the effects of strut thickness on the resorption and endurability of the HC scaffolds and the height and volume of newly augmented bone in the animal experiments.

The percentage of calcium ions released from HC300, which possessed the lowest porosity among the three HC scaffolds, was almost zero (0.27 ± 0.05%) after 38 days of immersion in the physiological saline (Fig. S2). In contrast, 16% of calcium ions was released from HC300 after 1 day of immersion in the weak acid solution, and afterward the percentage gradually increased. These results demonstrate that HC300 is stable in a physiological environment, while it is dissolved by exposure to weak acid, which is released from osteoclasts. Thus, HC300 seems to not be completely degraded in an *in vitro* physiological environment even after a few years.

The osteogenic differentiation of preosteoblastic cells (MC3T3-E1 cells) on HC300 was evaluated using an ALP (a marker of early osteoblast differentiation) activity assay and Alizarin Red staining, which allows the visualization of calcified deposition areas. The relative ALP activity increased from day 7 of cell incubation to day 14 (Fig. S3a). Furthermore, calcified deposition was clearly shown after 21 days of cell incubation (Fig. S3b). Thus, preosteoblastic cells differentiate into osteoblasts and achieve calcification in HC300.

### In vivo evaluations

At 4 and 12 weeks post-implantation (PI), HC100, HC200, and HC300 were extracted together with surrounding tissues. All three were entirely covered with tissues at 4 weeks PI (Fig. S4a–c). The thickness of the tissues covering these HC scaffolds increased during 8 weeks (between 4 and 12 weeks PI) (Fig. S4d–f).

**Fig. 5:**
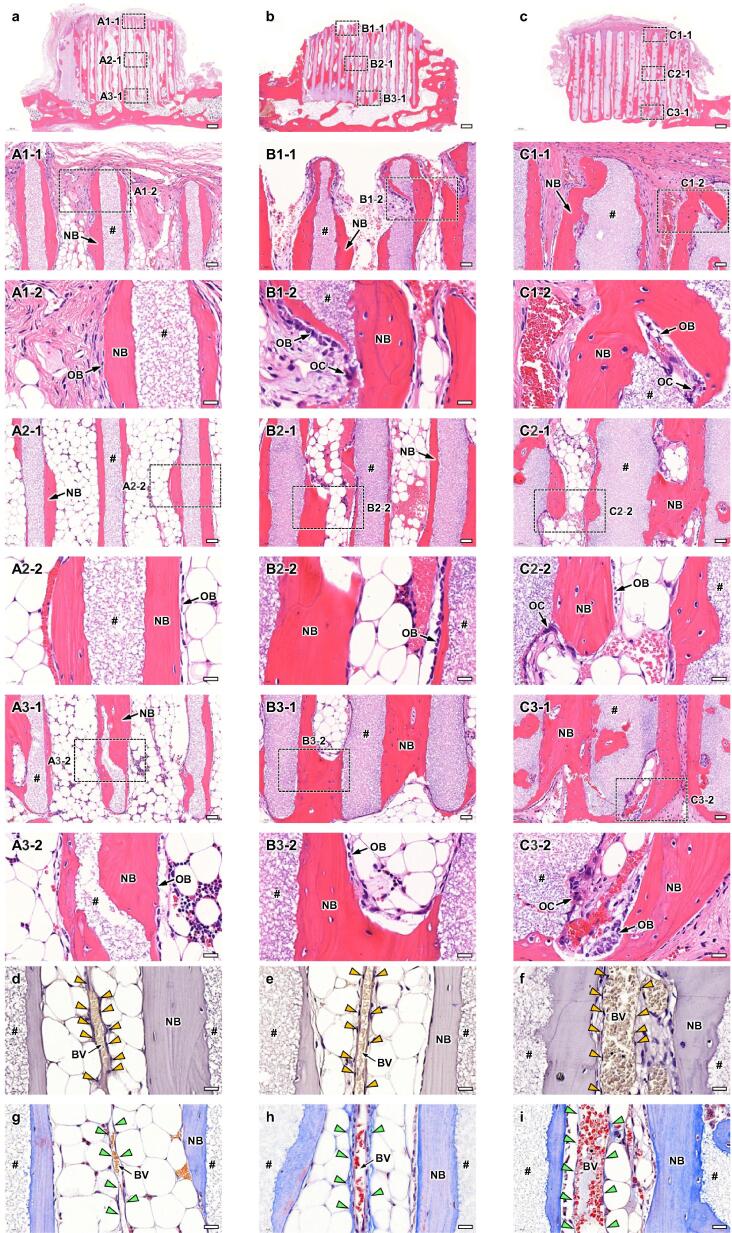
HE-stained sections at 12 weeks PI of HC100 (a), HC200 (b), and HC300 (c). Overviews (a-c); scale bars, 500 μm. Magnified images (A1-1-C1-1, A2-1-C2-1, and A3-1-C3-1); images of the regions A1-1-C1-1, A2-1-C2-1, and A3-1-C3-1 shown in images a-c; scale bars, 50 μm. Higher magnified images (A1-2-C1-2, A2-2-C2-2, and A3-2-C3-2); images of the regions A1-2-C1-2, A2-2-C2-2, and A3-2-C3-2 shown in images A1-1-C1-1, A2-1-C2-1, and A3-1-C3-1; scale bars, 20 μm. “#,” “NB,” “OB,” and “OC” indicate strut, new bone, osteoblast, and osteoclast, respectively. CD31-stained sections of HC100 (d), HC200 (e), and HC300 (f); scale bars: 20 μm. Yellow arrowheads indicate vascular endothelial cells. “BV” indicates blood vessel. MT-stained sections of HC100 (g), HC200 (h), and HC300 (i); scale bars: 20 μm. Green arrowheads indicate vascular endothelium.

At 4 weeks PI, μ-CT analysis showed that HC100, HC200, and HC300 maintained their original appearances (Fig. 3a–3c). The channels in all these HC scaffolds appeared whitish, indicating that new bone formed within the channels (Fig. 3a–3c). At 12 weeks PI, all these HC scaffolds were resorbed to varying degrees and the new bone regions increased in volume within the channels compared with that at 4 weeks PI (Fig. 3d–3f). The upper part of HC100 was deformed at 12 weeks PI; the height decreased and both side edges of the top surface were rounded (Fig. 3d). Additionally, the struts of HC100 at 12 weeks PI (Fig. 3d) became thinner compared with those at 4 weeks PI (Fig. 3a). In HC200, the top surface was partly dented and both side edges of the top surface were rounded at 12 weeks PI. Moreover, part of the struts was resorbed (Fig. 3e). In contrast with HC100 and HC200, HC300 maintained the original shape of the top surface at 12 weeks PI, although part of the struts was resorbed (Fig. 3f). To quantify the endurability to compression from the fasciae, compared with their original shapes (Fig. 3g), the deformation degrees of the HC scaffolds at 4 and 12 weeks PI were measured as follows: the angle of the side edge of the top surface (*θ*) and the minimum height (*H*_min_) were used as the deformation degree (Fig. 3h) [23]. All the HC scaffolds maintained a *θ* of approximately 90° at 4 weeks PI (Fig. 3i). However, the *θ* of HC100 and HC200 significantly increased to 124 ± 17° and 102 ± 18° at 12 weeks PI, respectively (Fig. 5i). In contrast, the *θ* of HC300 was 93 ± 7° at 12 weeks PI, which was not significantly different from that at 4 weeks PI (Fig. 3i). The *H*_min_ of HC100, HC200, and HC300 was 3.8 ± 0.3, 3.8 ± 0.3, and 3.9 ± 0.1 mm at 4 weeks PI, respectively (Fig. 3j), demonstrating that all these HC scaffolds almost maintained the original height (approximately 4 mm). At 12 weeks PI, the *H*_min_ of HC100 and HC200 was 2.5 ± 0.5 and 3.5 ± 0.6 mm, respectively (Fig. 3j); thus, their height significantly decreased after 8 weeks, that is, between 4 weeks PI and 12 weeks PI. In contrast, HC300 showed an *H*_min_ of 3.9 ± 0.1 mm at 12 weeks PI (Fig. 3j), demonstrating that it maintained the original height at 12 weeks after implantation. Additionally, the percent volume of remaining materials was defined by the ratio of the material volume (MV), that is, the volume of remaining struts to the total volume of the HC scaffold (TV), that is, the sum of channel volume and strut volume (Fig. 3g and 3 h). The MV/TV of HC100, HC200, and HC300 was 26.7 ± 6.4%, 58.0 ± 4.4%, and 73.7 ± 2.6% at 4 weeks PI and 18.7 ± 3.0%, 39.2 ± 8.5%, and 68.1 ± 0.7% at 12 weeks PI, respectively (Fig. 3k). Thus, all the HC scaffolds were resorbed during 8 weeks between 4 weeks PI and 12 weeks PI (Fig. 3k). Furthermore, significant differences in MV/TV were detected among these HC scaffolds at both 4 weeks PI and 12 weeks PI (Fig. 3k). The results of *θ*, *H*_min_, and MV/TV demonstrated that HC300 endured compression from the fasciae and was replaced by new bone while maintaining its original appearance, indicating that HC300 ensures the volume and shape of the new bone as intended.

Bone and blood vessels formed in the HC scaffolds were histologically analyzed. In all groups (Fig. 4a–4c), new bone had already reached the top edge of some struts at 4 weeks PI (Fig. 4A1-1, 4B1-1, and 4C1-1). Notably, in the group implanted with HC300, part of the top surfaces of the struts was covered with new bone (Fig. 4C1-1). In the regions around the top edges, osteoblasts resided on the new bone surface and blood vessels were present in the center of the channels (Fig. 4A1-2, 4B1-2, and 4C1-2). In the middle regions (Fig. 4A2-1, 4B2-1, and 4C2-1), new bone formed on the struts, osteoblasts resided on new bone, and blood vessels were formed in the channel centers in all groups (Fig. 4A2-2, 4B2-2, and 4C2-2). The findings in the lower regions were similar to those in the middle regions (Fig. 4A3-1, 4B3-1, and 4C3-1; 4A3-2, 4B3-2, and 4C3-2). The CD31-stained sections showed that vascular endothelial cells were arranged in the longitudinal direction of channels (Fig. 4d-f). In the MT-stained sections (Fig. 4g-i), the places where vascular endothelial cells resided were stained blue, indicating that collagen fibers in the vascular endothelium were stained. The distance between vascular endothelium portions can be regarded as the blood vessel diameter. The blood vessel diameter in HC300 was larger than that in HC100 and HC200 (Fig. 4d-i). Collagen fibers in the bone were also stained in the MT-stained sections (Fig. 4g-i).

At 12 weeks PI, both side edges on the top surface of HC100 and HC200 were rounded and the top surface was dented (Fig. 5a and 5b). In contrast, HC300 retained its original appearance, although a portion of the struts was resorbed and holes appeared in the struts (Fig. 5c). These findings were consistent with the μ-CT findings (Fig. 3). In all groups, new bone remained on the top edges of the remaining struts and blood vessels were present in the channels (Fig. 5A1-1, 5B1-1, and 5C1-1). Osteoblasts resided on the new bone in all groups (Fig. 5A1-2, 5B1-2, and 5C1-2). In contrast, osteoclasts resided in the dents of HC200 and HC300 struts, indicating that the struts underwent osteoclastic resorption (Fig. 5AB1-2 and 5C1-2). In the middle (Fig. 5A2-1, 5B2-1, 5C2-1, 5A2-2, 5B2-2, and 5C2-2) and lower (Fig. 5A3-1, 5B3-1, 5C3-1, 5A3-2, 5B3-2, and 5C3-2) regions, the histological findings were basically the same as those in the top regions. Overall, a larger number of osteoclasts seemed to reside in HC300 than in HC100 and HC200. The number of osteoclasts increased with increasing strut thickness. Vascular endothelial cells were stained by CD31 staining (Fig. 5d-f) and the collagen fibers in vascular endothelium were stained by MT (Fig. 5g-i). As at 4 weeks PI, blood vessels were arranged in the longitudinal direction of channels and the blood vessel diameter in HC300 was larger than that in HC100 and HC200 (Fig. 5d-i).

**Fig. 6:**
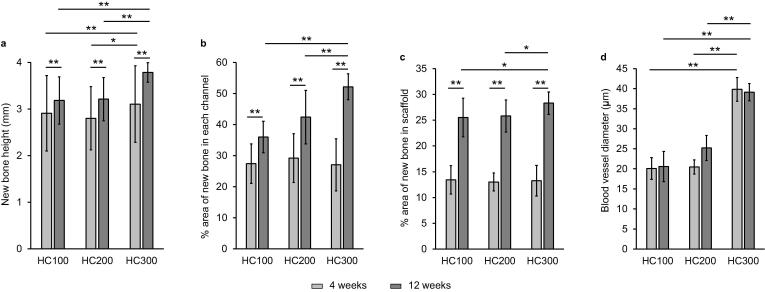
New bone height (a) and percent areas of new bone in each channel (b) and the entire scaffold (c) at 4 weeks and 12 weeks PI for HC100, HC200, and HC300. Diameter of blood vessels formed in HC100, HC200, and HC300 at 4 weeks and 12 weeks PI (d). *p < 0.05 and ^**^p < 0.01.

The height (Fig. 6a) and percent area of new bone (Fig. 6b) in each channel and the entire scaffold (Fig. 6c) were histologically analyzed from the HE-stained sections. New bone height (Fig. 6a) in HC300 at 4 weeks PI was 3.1 ± 0.8 mm, which was significantly greater than that in HC100 (2.9 ± 0.8 mm) and HC200 (2.8 ± 0.7 mm). In all groups, new bone height significantly increased during 8 weeks between 4 weeks PI and 12 weeks PI. At 12 weeks PI, new bone height in HC300 (3.8 ± 0.2 mm) was greater than that in HC100 (3.2 ± 0.5 mm) and HC200 (3.2 ± 0.5 mm). The percent area of new bone in each channel of HC100, HC200, and HC300 at 4 weeks PI was 27.4 ± 6.4%, 29.2 ± 7.9%, and 27.0 ± 8.4%, respectively (Fig. 6b). No significant difference in percent area of new bone at 4 weeks PI was detected among these HC scaffolds. At 12 weeks PI, the percent area of new bone in each channel of HC100, HC200, and HC300 was 36.0 ± 5.1%, 42.4 ± 8.6%, and 52.2 ± 4.2%, respectively. Thus, the percent area of new bone in each channel at 12 weeks PI significantly increased with increasing strut thickness. The percent area of new bone in the entire HC100, HC200, and HC300 scaffolds increased between 4 weeks PI and 12 weeks PI (Fig. 6c). Although the number of channels in HC300 was lower than that in HC100 and HC200, the new bone percentage in the entire HC300 scaffold at 12 weeks PI was 1.1-fold higher than that in HC100 and HC200 (Fig. 6c) because both the new bone height and area percentage in each channel in HC300 was higher than those in HC100 and HC200 (Fig. 6a and 6b). In contrast, the new bone percentage in the entire HC200 scaffold at 12 weeks PI was almost equal to that in HC100 (Fig. 6c) because the new bone height in HC200 was almost equal to that in HC100, although the new bone area percentage in each channel in HC200 was higher than that in HC100 (Fig. 6a and 6b). The above results demonstrated that, in all groups, new bone formation significantly progressed during 8 weeks between 4 weeks PI and 12 weeks PI. Notably, new bone augmentation in HC300 was considerably greater than that in HC100 and HC200. Furthermore, the blood vessel diameter in HC300 was ∼1.5–2-fold larger than that in HC100 and HC200 (Fig. 6d), which may be a reason why HC300 formed a larger amount of new bone than HC100 and HC200.

## Discussion

For achieving the predicted vertical bone augmentation using scaffolds, these should be replaced with new bone while bearing compression from the fasciae. To achieve this, this study provides a novel scaffold design that enables the compensation of the decrease in scaffold mechanical strength due to scaffold resorption with newly formed bone within the scaffold. This scaffold design synchronizes scaffold resorption and new bone formation, achieving vertical bone augmentation beyond previously reported strategies such as combinations of scaffold and growth factors, BMP, or stem cells.

Firstly, by comparing HC scaffolds with different strut thicknesses, we described the influence of strut thickness on scaffold resorption and endurability under the fasciae, and eventually on the height and volume of newly formed bone. The findings of the μ-CT and histological analyses in this study demonstrated that HC100, HC200, and HC300 bear compression from the fasciae and form almost equal amounts of new bone at 4 weeks PI. At 12 weeks PI, HC100 and HC200 were slightly rounded and shrank by compression from the fasciae. In contrast, HC300 bore the compression and retained its original appearance, even if a portion of the struts was resorbed. Furthermore, the resorbed regions were filled with new bone. Thus, HC300 is resorbed in harmony with new bone formation, complementing the reduction of scaffold strength. Consequently, HC300 grew new bone up to its original height and form, with 1.4- and 1.2-fold greater new bone volume than HC100 and HC200. Nevertheless, it should be noted that new bone augmented in HC100 and HC200 too. The fact that HC100 and HC200 were deformed despite new bone augmentation suggests that their bone mechanical strength immediately after formation is lower than that of natural bone. Therefore, scaffolds are required to maintain enough strength to bear the compression from the fasciae until new bone acquires a strength equal to natural bone. Considering that, the results of the present study show that a strut thickness ≥300 μm is necessary.

Secondly, we compared the endurability between our HC scaffolds and previously reported calcium phosphate-based scaffolds. In a previous similar study, calcium phosphate-based scaffolds were severely deformed until 4 weeks PI [23]. In that study, the scaffold deformation became exacerbated at 12 weeks PI; the *θ* was 126 ± 2° at 4 weeks PI and 136 ± 3° at 12 weeks PI [23]. Furthermore, the scaffold height decreased by approximately 20% during 8 weeks between 4 weeks PI and 12 weeks PI [23]. In contrast, in the present study, HC300 maintained the original *θ* (90°) and height even at 12 weeks PI. Owing to the superior endurability, HC300 leads the augmentation of bone with the predicted height and shape.

Next, we assessed the ability of the HC scaffolds to vertically augment bone through comparison with previous reports on the height and volume of newly formed bone using the same animal experiments as the present study, i.e., evaluation of vertical bone augmentation by implantation of scaffolds on the rabbit calvarium. For example, regarding the height of new bone, one study reported that calcium sulfate granules filled in rings augment new bone of 1.2 ± 0.7 and 2.6 ± 1.1 mm in height at 2 weeks and 8 weeks PI, respectively [48]. Moreover, they reported that when calcium sulfate granules are combined with dimethyloxalylglycine and sodium butyrate, which promote bone regeneration, the new bone heights at 2 weeks and 8 weeks PI reach 1.0 ± 0.3 and 3.2 ± 0.5 mm, respectively [48]. In another study, the uses of brushite, monetite, combined brushite and an anabolic conjugate drug, and combined monetite and an anabolic conjugate drug augment the maximum height of new bone by 0.8 ± 0.2, 1.4 ± 0.4, 1.7 ± 0.6, and 2.7 ± 0.5 mm, respectively, at 12 weeks PI [49]. Furthermore, at 4 weeks and 6 weeks PI of the combined hydroxyapatite blocks and recombinant human vascular endothelial growth factor (rhVEGF), the percent volume of new bone was 0.8 ± 0.6% and 0.8 ± 0.5%, respectively [27]. When collagen sponges containing recombinant human BMP-2 (rhBMP-2) in conjunction with β-TCP, biphasic calcium phosphate, bovine bone mineral, or blood clot are implanted, the percent amount of new bone at 14 weeks PI is 28.7 ± 4.6%, 31.9 ± 5.1%, 18.0 ± 2.2%, and 15.3 ± 2.9%, respectively [25]. Therefore, the single use of HC300 allowed rapid and abundant vertical bone augmentation compared to the use of various combined scaffolds and bone formation-promoting agents (VEGF, BMP, anabolic conjugate drug, dimethyloxalylglycine, and sodium butyrate). The outstanding ability of HC300 for vertical bone augmentation presumably results from its controlled scaffold structure (uniaxial channels, robust struts, and micropores in the struts) and analogous composition to bone mineral (i.e. carbonate apatite). Furthermore, even HC100 and HC200, which were inferior to HC300 in terms of their ability for vertical bone augmentation, are comparable to the combinations of scaffolds and augmenting agents, demonstrating that HC scaffolds are inherently appropriate for vertical bone augmentation.

From published literature and the present study, we can infer the required compressive strength of the scaffold for bearing compression from the fasciae in the present animal model. In our study, even HC100, which had the lowest compressive strength (11.1 ± 3.3 MPa) among the HC scaffolds, had maintained the original shape at 4 weeks after implantation. Thus, the maximum pressure from fasciae is less than ∼8 MPa, which is the minimum compressive strength of HC100 considering the standard deviation. Furthermore, Sheikh et al. reported that scaffolds with a compressive strength of ∼3 MPa collapsed within 4 weeks after implantation [50]. Although the compressive strength of the scaffold at 4 weeks after implantation is not described in the report by Sheikh et al., their results suggest that the initial compressive strength of the scaffold should be higher than ∼3 MPa.

Furthermore, the present study provides important findings on the correlation between scaffold structure and angiogenesis. In detail, HC300 formed larger-diameter blood vessels than HC100 and HC200. The following points should be noted: 1) all these HC scaffolds possessed basically the same chemical composition, channel aperture, and micropore volumes; 2) these HC scaffolds showed significant resorption only after 4 weeks PI; and 3) in contrast, the diameter of blood vessels formed in the HC scaffolds at 4 weeks PI were maintained up to 12 weeks PI. These facts indicate that ions released from the HC scaffolds were not related to blood vessel diameter. Additionally, the influence of channel aperture was also negligible, although previous reports revealed that channel aperture affects the delivery of oxygen and nutrients into the scaffold and the formation and diameter of blood vessels [37], [51]. Reportedly, the mechanical properties of the scaffold affect the response of endothelial cells to vascular endothelial growth factor [52] and the expression of growth factors in endothelial cells [53]. Furthermore, it has been reported that the differentiation of endothelial progenitor cells was proportional to the scaffold stiffness [54]. According to these findings, we can infer that the mechanical strength of the HC scaffold, which depends on the strut thickness, affects the diameter of blood vessels newly formed in the HC scaffold.

Finally, based on our present study and previous reports, we can presume that an optimum scaffold structure for vertical bone augmentation is an HC structure that possesses 300-μm-thick struts containing micropores and 230–300-μm-aperture channels. Furthermore, the present study showed that the strut thickness of the HC scaffolds considerably affected the diameter of blood vessels as well as new bone formation. Unfortunately, we were unable to clarify the mechanism by which strut thickness affects blood vessel diameter. Nevertheless, our findings are relevant for tissue regeneration. Therefore, we intend to unravel the mechanism in a future study.

## Conclusion

HC100, HC200, and HC300 all augmented new bone up to the top edge of some channels, resisted compression from the fasciae, and maintained their original appearances until 4 weeks PI. Afterward, HC100 and HC200 were resorbed and slightly shrank owing to compression from the fasciae. Although HC300 was also resorbed, its resorption rate coincided with new bone formation, allowing it to bear the compression from the fasciae and maintaining its original appearance even at 12 weeks PI. Furthermore, HC300 formed larger-diameter blood vessels than HC100 and HC200. Consequently, HC300 vertically augmented a larger bone, while being replaced by new bone, compared with HC100 and HC200. Nevertheless, the abilities of HC100 and HC200 to vertically augment bone were comparable to reported scaffolds combined with growth factors or other agents. Thus, HC scaffolds are inherently adapted for vertical bone augmentation.

## Compliance with Ethics Requirements

*All Institutional and National Guidelines for the care and use of animals were followed*.

## CRediT authorship contribution statement

**Koichiro Hayashi:** Conceptualization, Investigation, Resources, Writing – original draft, Visualization, Supervision, Project administration, Funding acquisition. **Masaya Shimabukuro:** Investigation. **Ryo Kishida:** Investigation. **Akira Tsuchiya:** Investigation. **Kunio Ishikawa:** Writing – review & editing, Funding acquisition.

## Declaration of Competing Interest

The authors declare that they have no known competing financial interests or personal relationships that could have appeared to influence the work reported in this paper.
