# Supplementary material for: Structurally optimized honeycomb scaffolds with outstanding ability for vertical bone augmentation
Source: J Adv Res. 2022 Jan 5;41:101–12. doi: 10.1016/j.jare.2021.12.010 (PMC9637481; doi:10.1016/j.jare.2021.12.010)
Supplement: Supplementary data 1 [file mmc1.docx]

Fig. S1. X-ray diffraction (XRD) patterns (a) and Fourier transform infrared (FT-IR) spectroscopy spectra (b) of HC100, HC200, and HC300. Hydroxyapatite was used as a reference.

Fig. S2. Percentages of calcium ions released from HC300 in physiological saline (pH = 7.4) and a weak acid solution (pH = 5.5) corresponding to the condition during osteoclastic resorption.

Fig. S3. (a) Relative ALP activity after 7 and 14 days of incubation of MC3T3-E1 cells on HC300. (b) Alizarin Red S stained-HC300 before seeding of MC3T3-E1 cells and after 21 days of cell incubation.

Fig. S4. Photographs of HC100 (a and d), HC200 (b and e), and HC300 (c and f) extracted at 4 weeks (a–c) and 12 weeks (d–f) after implantation.
